# Anti-Rods/Rings: A Human Model of Drug-Induced Autoantibody Generation

**DOI:** 10.3389/fimmu.2015.00041

**Published:** 2015-02-05

**Authors:** S. John Calise, Gerson D. Keppeke, Luis E. C. Andrade, Edward K. L. Chan

**Affiliations:** ^1^Department of Oral Biology, University of Florida, Gainesville, FL, USA; ^2^Rheumatology Division, Universidade Federal de São Paulo, São Paulo, Brazil; ^3^Fleury Medicine and Health Laboratories, Immunology Division, São Paulo, Brazil

**Keywords:** autoantibody, IMPDH2, hepatitis C, ribavirin, interferon, rods and rings

## Abstract

In recent years, autoantibodies targeting subcellular structures described as the rods and rings pattern in HEp-2 ANA have been presented as a unique case of autoantibody generation. These rod and ring structures (RR) are at least partially composed of inosine monophosphate dehydrogenase type 2 (IMPDH2), and their formation can be induced *in vitro* by several small-molecule inhibitors, including some IMPDH2 inhibitors. Autoantibodies targeting these relatively unknown structures have been almost exclusively observed in hepatitis C virus (HCV) patients who have undergone treatment with pegylated interferon-α/ribavirin (IFN/RBV) combination therapy. To date, anti-RR antibodies have not been found in treatment-naïve HCV patients or in patients from any other disease groups, with few reported exceptions. Here, we describe recent advances in characterizing the RR structure and the strong association between anti-RR antibody response and HCV patients treated with IFN/RBV, detailing why anti-RR can be considered a human model of drug-induced autoantibody generation.

## Introduction

Autoantibodies targeting rods and rings (RR) have become an intriguing topic of discussion since 2005, when the unique RR pattern was first observed in a standard IIF-HEp-2 assay. Patient sera presenting this novel staining pattern were recognized to be hepatitis C virus (HCV) patients treated with the typical pegylated interferon-α/ribavirin combination therapy (IFN/RBV). Historically, HCV infection has been loosely associated with a collection of different types of autoantibodies, including antinuclear, anti-smooth muscle, and anti-liver/kidney microsome antibodies, among many others. The production of anti-rods/rings (anti-RR) antibodies appears to be distinct from current knowledge of typical autoantibody generation in HCV patients. There are presently a few hypotheses attempting to explain how antibodies targeting self-proteins are generated, including suggestions that apoptotic bodies, epigenetic modifications, or cross-reactivity between self-proteins and foreign proteins, known as molecular mimicry, may play a role in autoantibody generation (1). Anti-RR antibodies seem to have an additional stipulation that has been reported by several independent laboratories: treatment with IFN/RBV is, with few exceptions out of all patients reported to date, required to observe anti-RR seropositivity; anti-RR antibodies have not been observed in any HCV patients prior to treatment (2–6). This implies that anti-RR antibodies should be considered drug-induced autoantibodies, similar to previously documented cases of antinuclear and anti-histone antibodies in drug-induced lupus erythematosus, which has been demonstrated in both humans and mice (7). The story of anti-RR antibodies becomes more compelling when one considers that when the RR ANA pattern was first observed, the antigenic filamentous and annular structures targeted by these antibodies were completely unfamiliar to the field. In recent years, our laboratory and other laboratories, who stumbled upon the same structure in mammalian cells through different means, have begun to elucidate the structural and functional characteristics of these previously unknown autoantigens (8–11). In the following sections, we present anti-RR antibodies as a human model for drug-induced autoantibody generation, as well as report recent findings on the unusual enzymatic polymers known as RR that are targeted by these antibodies.

## Autoantigenic Rod and Ring Structures

**Figure 1:**
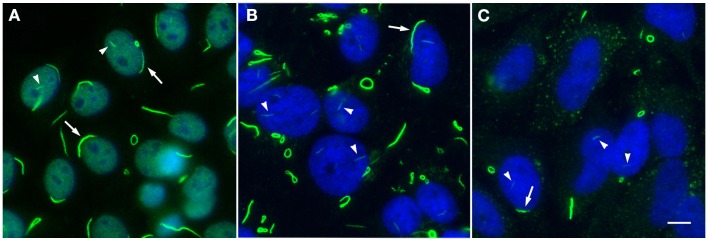
**Rods and rings induced under various conditions exhibit similar phenotypic patterns**. **(A)** Autoantibodies in prototype anti-RR serum 604 from a hepatitis C patient (green, DyLight 488 donkey anti-human IgG) recognize RR structures in a standard HEp-2 ANA screening; nuclei are counterstained with DAPI (blue). Serum 604 also shows fine nuclear speckled staining. **(B)** RR induced by 24 h treatment with 1 mM ribavirin in HeLa cells are detected by another human prototype hepatitis C serum It2006 (green, Alexa Fluor 488 goat anti-human IgG); nuclei counterstained with DAPI (blue). **(C)** HeLa cells deprived of glutamine for 48 h exhibit RR in ~50% of cells and are recognized by serum It2006 (green, Alexa Fluor 488 goat anti-human IgG); nuclei counterstained with DAPI (blue). Under each condition, nuclear rods (arrowheads) can be clearly visualized alongside more conspicuous cytoplasmic rods and rings, which typically appear much longer and thicker than their nuclear counterparts; rings can also be found in the nucleus, although this is a less common observation. Additionally, while cytoplasmic RR appear to be more common than nuclear RR, rods often localize to the perinuclear region (arrows). All panels are shown at 200× magnification. Scale bar: 10 μm.

In order to properly discuss anti-RR as a human model of autoantibody generation, it is necessary to first present current knowledge of the novel targets of these antibodies, since the process of elucidation of the RR structures has only been ongoing for less than a decade. Morphologically, RR structures present themselves in two major forms, discrete filamentous “rods” 3–10 μm in length or annular “rings” 2–5 μm in diameter (Figure 1). Both forms are observed primarily in the cytoplasm, although generally smaller structures are regularly found in the nucleus under cellular conditions allowing for RR formation (5). While it has been shown that RR are not associated with any known organelles, we have observed perinuclear rods that appear to wrap around or position along the cytoplasmic side of the nuclear membrane (2, 8). The primary target and major component of the RR structures is the enzyme inosine monophosphate dehydrogenase type 2 (IMPDH2), which functions primarily in the GTP biosynthesis pathway by catalyzing the rate-limiting conversion of inosine monophosphate into xanthosine monophosphate. IMPDH2 and its other isoform IMPDH1 share 84% sequence identity, but while several laboratories have identified IMPDH2 as the main contributor to formation of RR, none have yet been able to definitively determine the contribution of IMPDH1 (2, 4, 8–11). Since anti-RR antibodies were first observed in the sera of HCV patients treated with IFN/RBV, both interferon-α and ribavirin were tested *in vitro* to determine their effects on cultured cells; while IFN had no effect on RR formation, the IMPDH inhibitor ribavirin induced RR formation in >95% of cultured cells (2, 8, 11). Previous reports showed that another IMPDH inhibitor, mycophenolic acid, induced RR in a high percentage of cultured cells (Table 1) (10, 11).

**Table 1:** **Associations between RR-inducing inhibitors and anti-RR production**.

Inhibitor	Mode of action	Clinical use	Anti-RR in patients?	Major references
Acivicin	Glutamine analog	N/A (experimental antitumor agent)	None reported	(8)
Decoyinine	Nucleoside analog	N/A (experimental antitumor agent)	None reported	(11)
DON	Glutamine analog	N/A (experimental antitumor agent)	None reported	(8)
Mycophenolic acid	Inhibits IMPDH	Prevention of organ transplant rejection; lupus nephritis	None reported	(10)
Pemetrexed	Folate antimetabolite	Pleural mesothelioma; non-small cell lung cancer	None reported	(5)
Ribavirin	Inhibits IMPDH	Hepatitis C; human respiratory syncytial virus	Yes, 20–38% of HCV patients^a^	(2, 3)

DON, 6-diazo-5-oxo-l-norleucine; IMPDH, inosine monophosphate dehydrogenase;

*^a^anti-RR observed in 20–38% of hepatitis C patients treated with pegylated interferon-α/ribavirin combination therapy*.

A previous study from our laboratory also identified cytidine triphosphate synthetase type 1 (CTPS1) as an additional component of RR (8). CTPS, which also has two isoforms with 74% sequence identity, is responsible for the rate-limiting conversion of uridine triphosphate to cytidine triphosphate. Two glutamine analogs, 6-diazo-5-oxo-l-norleucine (DON) and acivicin, which non-specifically inhibit CTPS by nature of their similarity to glutamine, have also been reported to induce RR formation *in vitro* (8). Curiously, despite the existence of several drugs that can induce RR formation *in vitro*, IFN/RBV-treated HCV patients are the only patients we have seen develop antibodies targeting these structures to date (Table 1). There has been some debate over the past couple of years regarding the contribution of CTPS to RR formation. Since our 2011 study that identified CTPS as a potential component of RR using an antibody targeting *Drosophila* CTPS, we have been unable to validate the presence of CTPS in RR with any commercially available CTPS antibodies (5). Additionally, we have yet to observe any patient sera that react with CTPS, despite demonstrating that a number of anti-RR sera react positively with IMPDH (6, 12). Certainly, we are still in the early stages of fully expounding the structural details of RR, and we may yet find a more definitive link between CTPS and IMPDH by way of this unique structure.

Although the study of RR *in vitro* was initiated using small-molecule inhibitors to induce structure formation, RR have been observed in other circumstances in the absence of these inhibitors. Certain non-human cell lines have been found to continuously express RR without treatment with inhibitors, such as normal rat kidney epithelial cells (NRK), male rat kangaroo kidney epithelial cells (Ptk2), primary mouse fibroblasts (3T3), mouse leukemic monocyte/macrophage cells (RAW264.7), and Chinese hamster ovary cells (CHO) (5, 6). These various cell lines present RR in anywhere from 10 to 80% of cells, depending on the cell type. The most notable and impressive example is the steady occurrence of RR in undifferentiated mouse embryonic stem cells; the typical observed ratio of 9:1 rods to rings is reversed in this cell type, which show a 9:1 rings to rods ratio (8). While it is not uncommon for mouse cell lines to present RR without apparent manipulation or induction of the structures, this unusual ratio implies that the structures in these cells have the potential to be structurally or functionally different. Recent work from our laboratory points to another method of inducing these structures through glutamine deprivation (9). In that study, HeLa cells deprived of glutamine for at least 48 h developed RR in ~50% of cells; the percentage of cells presenting RR increased to ~98% (similar to IMPDH inhibitors) when depletion of exogenous glutamine was combined with treatment of methionine sulfoximine, a glutamine synthetase inhibitor. These reported phenotypic differences in RR expression between cells treated with inhibitors, cells presenting RR without extrinsic manipulation, and cells deprived of glutamine suggest that functionality of the structures may vary depending on cellular conditions, although at this time we can only speculate on functional differences because no direct evidence has been reported yet. At least, the presence of IMPDH2 and reactivity with prototype anti-RR sera has been validated in all forms of RR observed in mammalian cells to date, so it can be concluded that we are observing the same structures under all of these conditions.

## Exclusivity of Anti-RR in IFN/RBV-Treated HCV Patients

While only a few studies have been completed to date examining the prevalence of anti-RR positivity in HCV and other disease cohorts, a handful of common trends in the data have already emerged. In 2011, one of the first reports on anti-RR came out that used radioimmunoprecipitation (RIPA) to analyze the prevalence of anti-IMPDH2 antibodies in various disease groups; using this technique, they reported the presence of anti-IMPDH2 in 35.2% of HCV-RNA carriers (*n* = 108), compared to 31% of anti-actin positive patients suspected of autoimmune hepatitis (*n* = 42), 5% of hospitalized HCV-RNA negative patients (*n* = 100), 6.2% of HBV-DNA positive patients (*n* = 113), 13.7% of antinuclear antibody positive patients (*n* = 51), 3.2% of anti-mitochondrial positive (type M2) patients suspected of primary biliary cirrhosis (*n* = 31), and 2% of healthy blood donors (*n* = 100) (13). Although anti-RR is not exactly the same as anti-IMPDH2, we can speculate that this increased prevalence of anti-IMPDH2 in HCV-RNA carriers could be related to IFN/RBV therapy potentially altering the immunogenicity of IMPDH2 protein. In early 2012, another study reported clinical relevance of anti-RR in a cohort of 75 IFN/RBV-treated HCV patients from Italy (2). Although some preliminary work was presented on some small cohorts of patients prior to this publication, this study resulted in the first indications of general trends seen with the anti-RR pattern to date. Despite the fact that extensive work on HCV has been published over the last few decades, it was not until recently that the anti-RR pattern was observed. This may have been largely due to the fact that only a few commercial HEp-2 substrate slides have been shown to detect anti-RR to date, those from INOVA Diagnostics (San Diego, CA, USA) or Euroimmun (Lübeck, Germany), for example. For reasons still unknown, the cells in these slides contain the RR structures necessary to detect anti-RR, while the cells in slides from many other companies do not. In this study by Covini et al., substrate from INOVA Diagnostics led to the observation that 15 out of 75 HCV patients (20%) who received IFN/RBV treatment were positive for these anti-RR antibodies reacting with these unique filamentous RR structures (2). This observation was especially noteworthy since anti-RR were not detected in the sera of the same patients that were collected prior to antiviral therapy, and these antibodies were not detected in sera of patients from any other control groups, which included 105 primary biliary cirrhosis, 43 primary sclerosing cholangitis, 56 autoimmune hepatitis, 100 untreated hepatitis B-related chronic active hepatitis, and 100 hepatocellular carcinoma patients, as well as 100 blood donors. Additionally, the authors noted that patients who did not respond to therapy or relapsed were significantly more likely to be anti-RR positive (10/30, 33%) than patients who responded well to IFN/RBV therapy (5/45, 11%). Although this study has been succeeded by additional work on other patient cohorts, some salient hypotheses made in the discussion have yet to be answered.

While there are some well-known cases in the field of hepatology of drugs inducing autoantigens, those reported thus far are organ-specific responses. RR are the first reported case of induction of an autoantigen related to IFN/RBV treatment in HCV patients. In the same study from Covini et al., they showed that *in vitro* treatment of cell lines with RBV induced the formation of RR structures, leading them to hypothesize that RBV could be inducing structure formation *in vivo* as well, perhaps in hepatocytes during the process of antiviral therapy, which would eventually lead to an immune response in the form of anti-RR. An additional consideration is that the exclusivity of the association of anti-RR with treated HCV patients suggests a potential for a biological role for the RR structures, since in this initial study, anti-RR were significantly more prevalent in non-responders and relapsers than in patients responding to IFN/RBV therapy. While it is still not known why RR structures appear to become antigenic in HCV-IFN/RBV patients but not in patients treated with other drugs known to induce these structures *in vitro*, it is at least striking that RBV, an inhibitor of IMPDH, may be causing rearrangement of the same enzyme into a structure presented to the immune system as antigenic.

Although the study from Covini et al. reported that patients in their cohort who did not respond to therapy or relapsed were significantly more likely to be anti-RR positive, results from a follow-up study comparing the anti-RR titers and serum reactivity with anti-IMDPH2 between two new patient cohorts led to some unexpected results (4). In one cohort of 46 Italian HCV patients positive for anti-RR, significantly higher titers were found in patients who relapsed from therapy compared to non-responding (*p* = 0.004) or responding (*p* = 0.015) patients. Patients from a US cohort (*n* = 47) who did not respond or relapsed had significantly higher titers than responding patients (*p* = 0.0016). Analysis of the two cohorts using RIPA showed that anti-RR antibodies were primarily anti-IMPDH2 in 96% of patients in the Italian cohort, compared to only 53% in the US cohort; this is a strong indication that the RR structures are composed of other unknown proteins that may become antigenic in certain patients. It should be noted that no laboratories have determined any anti-RR positive patient sera to be reactive with CTPS to date (4, 6, 12). A longitudinal study published just after this report confirmed the previous trend observed in the Covini et al. paper, reporting that 38% of 108 treated HCV patients were positive for anti-RR while none of the 166 untreated HCV patients or patients receiving only interferon-α (*n* = 23) or only ribavirin (*n* = 3) presented positive anti-RR staining (3). Notably, they also reported that anti-RR positivity appeared after 3–6 months of treatment and remained in a percentage of Brazilian IFN/RBV-treated HCV patients 6–12 months post-treatment. Another publication with the Brazilian HCV cohort addressing the behavior of anti-RR antibody production during IFN/RBV treatment shows that the temporal kinetics of the humoral autoimmune response to IMPDH2 resembled that of a conventional humoral response to infectious agents regarding titer, avidity maturation, and isotype levels, but showed a considerably slower pace in titer increase and avidity maturation, as well as in isotype class switch (14). Yet another study from 2013 once again validated the apparent exclusivity of anti-RR to IFN/RBV-treated HCV patients; in this study, anti-RR antibodies were not observed in any primary biliary cirrhosis or systemic lupus erythematosus sera, and prior treatment with IFN/RBV was the only independent predictor of anti-RR positivity in a cohort of Canadian patients (6). Although they reported that only 5% of their patient cohort was positive for anti-RR, they acknowledged that the large majority of their patients were treatment-naïve, which explains the lower percentage compared to previous studies. Either way, there are clearly a few independent data sets from varying geographic regions of the world that support the hypothesis that anti-RR antibodies are a model for drug-induced autoantibody generation.

Despite the evidence supporting the exclusivity of anti-RR with IFN/RBV treatment, certainly some rare exceptions have been reported where anti-RR antibodies have been observed in patients naïve to IFN/RBV. The most notable exception to the premise of exclusivity lies in the results from the National Health and Nutrition Examination Survey (NHANES), where 0.8% of 4,754 individuals from the US, designed to be representative of the general US population, were reported anti-RR positive (15). Interestingly, anti-RR titers in the NHANES patients were measured to be significantly lower (median = 1:320) than the titers in the US and Italian HCV cohorts from Carcamo et al. (medians = 1:1600 and 1:25,600, respectively), a potential sign that low-titer anti-RR may occur rarely in the general population and may not be associated with a certain disease or treatment, while high-titer anti-RR may be associated with IFN/RBV (15). To date, there have also been two individuals with no known association with ribavirin reported to be positive for anti-RR, one hepatitis B patient and one systemic lupus erythematosus patient; the implications of these two patients’ reactivity with RR have yet to be determined (3, 15). However, with current evidence, we can conclude that in a vast majority of cases, anti-RR antibodies are likely to be related to IFN/RBV treatment and could therefore be considered a unique model for drug-induced autoantibody generation.

## Conclusion

Rods and rings and the autoantibodies that target these structures have become a major interest for several laboratories around the world. When some of the more surprising data about this antibody/antigen relationship are considered, it is easy to see the potential for anti-RR to develop as a truly unique example of autoantibody generation in humans. For example, it is not often that titers up to 1:819,200 are reported in literature (4), and it is not often that subcellular structures as large as RR go unnoticed for so long. On top of that, IMPDH is a vital enzyme for proliferation and viability in the cells of diverse species, and one can see the potential for these structures to play crucial roles in homeostasis and metabolism, as has been suggested in recent studies (8–11). For now, the study of the possible clinical impact of these antibodies and the study of the composition and function of the RR structures must continue, with the hope that the clinical and basic science aspects of this system will eventually be bridged to help improve the lives of patients and to bolster our knowledge of large, potentially immunogenic enzymatic aggregates in the cell. Since high-titer anti-rods/rings autoantibodies appear to be somewhat exclusive to IFN/RBV-treated HCV patients, it is reasonable to hypothesize that the combination of IFN/RBV and HCV creates a unique situation in which HCV gene products have a special affinity for RR structures. This putative RR/HCV structure could become autoantigenic and lead to production of autoantibodies, especially under the influence of IFN. It is likely that many or all of the drugs listed in Table 1 could induce RR *in vivo*, but perhaps an exceptional interaction with HCV enables RR to present as autoantigenic in a subset of HCV patients that cannot occur in other diseases.

## Conflict of Interest Statement

The authors declare that the research was conducted in the absence of any commercial or financial relationships that could be construed as a potential conflict of interest.

## Abbreviations

ANA, antinuclear antibody; CTPS1, cytidine triphosphate synthetase 1; DON, 6-diazo-5-oxo-l-norleucine; HCV, hepatitis C virus; IFN/RBV, pegylated interferon-α/ribavirin therapy; IMPDH2, inosine monophosphate dehydrogenase 2; RIPA, radioimmunoprecipitation assay; RR, rods and rings.
